# Association between Depressive Symptoms and Metabolic Syndrome in Police Officers: Results from Two Cross-Sectional Studies

**DOI:** 10.1155/2012/861219

**Published:** 2012-01-18

**Authors:** Tara A. Hartley, Sarah S. Knox, Desta Fekedulegn, Celestina Barbosa-Leiker, John M. Violanti, Michael E. Andrew, Cecil M. Burchfiel

**Affiliations:** ^1^Biostatistics and Epidemiology Branch, Health Effects Laboratory Division, National Institute for Occupational Safety and Health, Morgantown, WV 26505, USA; ^2^Department of Community Medicine, School of Medicine, West Virginia University, Morgantown, WV 26506, USA; ^3^College of Nursing, Washington State University, Spokane, WA 99210, USA; ^4^Department of Social and Preventive Medicine, School of Public Health and Health Professions, State University of New York at Buffalo, Buffalo, NY 14214, USA

## Abstract

Policing is one of the most dangerous and stressful occupations and such stress can have deleterious effects on health. The purpose of this study was to examine the association between depressive symptoms and metabolic syndrome (MetSyn) in male and female police officers from two study populations, Buffalo, NY and Spokane, WA. Depressive symptoms were measured using the Center for Epidemiologic Studies-Depression (CES-D) scale. MetSyn was defined using the 2005 AHA/NHBLI guidelines. Analysis of covariance was used to describe differences in number of MetSyn components across depressive symptom categories. The number of MetSyn components increased significantly across categories of CES-D for Spokane men only (p-trend = 0.003). For each 5-unit increase in CES-D score, odds increased by 47.6% for having hypertriglyceridemia, by 51.8% for having hypertension, and by 56.7% for having glucose intolerance. Exploring this association is important since both are predictors of future chronic health problems and the results could be helpful in developing future gender-specific prevention and intervention efforts among police officers.

## 1. Introduction

Police officers are members of a unique occupational population whose exposure to potentially traumatic and life threatening events is part of their professional duty [1]. Additionally, officers routinely encounter organizational stressors, such as court appearances, excessive paperwork, and shift work [1]. Higher levels of perceived work stress have been associated with depression among older police officers [2]. Police officers have more adverse cardiovascular disease (CVD) risk factor levels and higher rates of CVD morbidity and mortality compared to other populations [3–6]. Work stress and depression are risk factors for the development of CVD and diabetes [2, 7–20].

The MetSyn is a clustering of abnormalities (abdominal obesity, hypertension, dyslipidemia, glucose intolerance) that has been associated with increased risk for subclinical atherosclerosis, CVD, diabetes, target organ damage, and total mortality [21]. The association between depression and the MetSyn has been studied over the past decade [8, 9, 11, 13, 14, 18, 19, 22–29], and more recently it has been suggested that MetSyn may be intermediate in the pathway between depression and CVD [11, 13, 15, 18, 19, 30, 31]. Multiple pathophysiological mechanisms underlying the association between depression and MetSyn have been proposed. One mechanism is neuroendocrine and involves overstimulation of the hypothalamic-pituitary adrenal (HPA) axis which leads to excess cortisol secretion and has been associated with both the development of depression and the MetSyn components [32, 33]. Another indirect pathway is through adverse health behaviors associated with depression, such as cigarette smoking, excessive use of alcohol, and a sedentary lifestyle, which may be used as coping responses and subsequently increase risk of MetSyn and CVD [8, 11, 19].

The association between depression and MetSyn appears to be stronger in women, although the explanation for this gender difference is somewhat unclear [11, 15, 18, 23, 25, 26]. One possible explanation involves the role of sex hormones. Overactivation of the HPA axis due to stress or depression may result in decreased levels of sex hormones like estrogen [34]. Decreased levels of estrogen, such as that occurs during menopause, can elevate visceral fat mass, a component of MetSyn [34].

Police officers are considered to be a relatively young and presumably healthy occupational group (i.e., healthy worker effect); yet they suffer from high levels of stress and CVD. The purpose of this study was to examine the association between depressive symptoms and the MetSyn separately among male and female police officers from two cross-sectional studies. No known studies were identified which examined this association in police officers. Participants are police officers from two geographically different locations, which may make the findings more generalizable to other groups of police officers. Based on the literature, the specific study hypotheses were the following: (1) depressive symptoms would be associated with a larger number of MetSyn components in male and female police officers, (2) the association of depressive symptoms with MetSyn and its components would differ between male and female police officers, and (3) the association between depressive symptoms and MetSyn would not differ by geographic location.

## 2. Methods

Participants included those from two studies at two separate locations: Buffalo, New York and Spokane, Washington.

### 2.1. Buffalo Cardio-Metabolic Occupational Police Stress (BCOPS) Study

The Buffalo Cardio-Metabolic Occupational Police Stress (BCOPS) Study, a cross-sectional study of urban police officers, was conducted between 2004 and 2009. The purpose of the BCOPS Study was to examine the association between police stressors and physiological and psychological health consequences. The Center for Health Research at the School of Public Health and Health Professions, State University of New York in Buffalo, NY served as the data collection site. All 710 active duty police officers from the Buffalo, NY, Police Department were invited to participate and the recruitment was ongoing for five years. No specific inclusion criteria were used for the study, other than that the participant be a sworn police officer and willing to participate in the study. Women officers who were pregnant at the time of examination were excluded (*n* = 2). All participants provided informed consent and all phases, testing, and reports of the study were approved by the State University of New York at Buffalo Internal Review Board and the National Institute for Occupational Safety and Health Human Subjects Review Board. Of the 464 officers examined, 54 were removed from analyses (33 retired, 1 missing demographic information, 3 missing depressive symptoms, 17 missing MetSyn) leaving a final sample of 410 officers (304 men, 106 women; age range 21–66).

Questionnaires were administered to collect demographic and lifestyle information and psychosocial measures. Participants provided a medical history (including history of CVD) and a 12-hour fasting blood sample was collected by a certified phlebotomist. Medication use was ascertained through self-report and by inventory of current medications brought to the clinic. Blood parameters for the MetSyn were measured by standard laboratory techniques on the Beckman Coulter LX20 clinical chemistry analyzer and included a blood lipid panel for high-density lipoprotein cholesterol (HDL-C) and triglycerides and chemistry panels for glucose [35]. Anthropometric measures were conducted by trained clinic personnel. Waist circumference was measured as abdominal girth at the highest point of the iliac crest and the lowest point of the costal margin in the mid-axillary line. Blood pressure was determined using the average of the second and third of three separate measurements of resting systolic and diastolic blood pressure obtained with a standard sphygmomanometer.

### 2.2. Spokane Heart Study (SHS)

The Spokane Heart Study (SHS) was a prospective, longitudinal study investigating the natural history of preclinical atherosclerosis. Details of the study have previously been reported [4]. Over a 12-year period, over 1,000 asymptomatic men and women were enrolled and followed every two years, generating an extensive data set of medical and lifestyle histories, blood and urine measurements, and Electron Beam Computed Tomography (EBCT) measures of coronary artery calcification. At baseline, all participants completed a comprehensive questionnaire which measured key behavioral, social, and lifestyle domains. Fasting blood samples and anthropometric measures were taken prior to the EBCT scan. Routine assays for blood chemistries, lipid profile, and hematology were performed [4]. The sample was almost equally divided between men and women. All participants provided informed consent and all phases, testing, and reports of the study were approved by the Washington State University Institutional Review Board. For the current study, data collection years 2003–2006, years most comparable to the BCOPS study, were used. Based on the questions, “what is the name of your current employer” and “what is your current job title,” 134 police officers were identified. Of the 134 officers participating in the study, four were removed from analyses (two missing depressive symptoms and two missing MetSyn) leaving a final sample of 130 Spokane police officers (121 men, 9 women; age range 35–62).

### 2.3. Depressive Symptoms

In both studies, depressive symptoms were measured using the Center for Epidemiologic Studies-Depression (CES-D) scale. The CES-D is a short 20-item scale designed to measure symptoms of depression (e.g., poor appetite, restless sleep, sadness) in the general population on a 4-point scale [36]. The 4-point scale represents how much each symptom occurred during the past seven days as follows: 0 (rarely or none of the time, less than 1 day), 1 (some or little of the time, 1-2 days), 2 (occasionally or a moderate amount of time, 3-4 days), and 3 (most or all of the time, 5–7 days). The CES-D is scored by reverse coding the appropriate items and summing the scores to obtain an overall score. Respondents with scores between 0 and 15 are unlikely to be clinically depressed, scores of 16 to 21 indicate mild to moderate depression, and scores of 22 or greater are associated with major depression [36]. The CES-D has acceptable reliability, Cronbach alpha of 0.85, and a split-half reliability ranging from 0.76 to 0.85 [34]. For the current study, the continuous CES-D and three categories of CES-D were used: low (0–8), middle (9–15), and high (16 or greater).

### 2.4. MetSyn

The MetSyn criteria were based on the National Cholesterol Education Program Adult Treatment Panel guidelines with recent modifications from the American Heart Association and the National Heart, Lung, and Blood Institute [37]. The individual MetSyn components included the following: (1) abdominal obesity (gender-specific waist circumference ≥102 cm in males, ≥88 cm in females), (2) hypertension (systolic blood pressure ≥ 130 mm Hg, diastolic blood pressure ≥ 85 mm Hg, or reported physician-diagnosed hypertension and antihypertensive treatment), (3) reduced HDL-C (gender-specific fasting HDL-C <40 mg/dL in men, <50 mg/dL in women, or reported treatment with nicotinic acid or fibrates), (4) elevated triglycerides (fasting triglycerides ≥ 150 mg/dL, or reported treatment with nicotinic acid or fibrates), and (5) glucose intolerance (fasting serum glucose ≥ 100 mg/dL, or reported treatment for diabetes). Participants were categorized according to the number of MetSyn components (0–5). MetSyn was considered present in individuals with three or more components.

### 2.5. Statistical Methods

Descriptive statistics were used to characterize the study population. The number of MetSyn components and the prevalence of MetSyn and each individual component were calculated. Analyses of variance and covariance were used to estimate and describe the unadjusted and multivariate adjusted mean count of MetSyn components across CES-D categories. Tests for trend were obtained from linear regression analyses with continuous CES-D as the independent variables and count of MetSyn components as the dependent variable. Multivariate models were used to adjust for age, smoking status, and marital status. These covariates were chosen based on their association with depression and MetSyn and evidence in the literature. Logistic regression was used to calculate the odds for having each of the five MetSyn components by categories of CES-D score. Even though the tests of interaction for gender and location (Buffalo, Spokane) were not statistically significant, results were stratified in order to provide comparisons between locations and between male and female officers. All analyses were conducted using the SAS software, Version 9.2 (SAS Institute, Cary NC). Due to the small number of Spokane women in this study, the primary analysis of the association between CES-D and MetSyn could not be conducted in this group; however, descriptive statistics for these nine participants are reported.

## 3. Results

### 3.1. Demographic Characteristics

Demographic characteristics of the Buffalo, NY and Spokane, WA police officers are shown in Table 1. The Spokane officers were between six and eight years older than the Buffalo officers (Spokane range = 35–62, Buffalo range = 21–66) and nearly all white (1/130) compared to approximately 22% of Buffalo officers who were black or Hispanic. More Spokane officers had completed at least four years of college (44% versus 34%) and the Spokane men held higher ranks (above police officer) than the Buffalo men or women (67.7% versus 30.5% and 19.8%, resp.). More male officers were married (78.2% for Buffalo men, 91.7% for Spokane men) than female officers (58.5% for Buffalo women, 55.6% for Spokane women). More female Buffalo officers were current smokers compared to the other three groups. The mean (SD) CES-D score was 7.5 (6.6) for Buffalo men, 6.6 (5.5) for Spokane men, 8.7 (8.2) for Buffalo women, and 5.8 (4.5) for Spokane women.

### 3.2. MetSyn and Its Components

The prevalence of each of the MetSyn components tended to be higher for Spokane officers compared to Buffalo officers (Table 2). The most notable differences were the higher prevalence for abdominal obesity, elevated triglycerides, and glucose intolerance in Spokane women compared to Buffalo women. The differences were not as large between Spokane and Buffalo men. The mean values for each of the five MetSyn components were higher for Spokane officers compared to Buffalo officers, most notably triglyceride levels for Spokane women (165.3 mg/dL) compared to Buffalo women (88.4 mg/dL). The prevalence of MetSyn (≥3 components) was highest among Spokane officers, 37.2% for men and 33.3% for women, compared to Buffalo officers, 31.6% for men and 8.5% for women—half of the Buffalo women did not have any of the MetSyn components. Accordingly, the number of MetSyn components was higher among Spokane officers, 2.1 (1.4) for men and 1.7 (1.9) for women, compared to Buffalo officers, 1.9 (1.4) for men and 0.9 (1.1) for women.

### 3.3. Association between Number of MetSyn Components and Categories of CES-D Score

The unadjusted number of MetSyn components increased significantly across categories of CES-D for Spokane men (CES-D score 0–8 = 1.9 (1.3), 9–15 = 2.6 (1.3), ≥16 = 3.1 (1.6), p-trend = 0.003) (Table 3). This association remained significant after adjustment for age (p-trend = 0.004) and multivariate adjustment for age, smoking, and marital status (p-trend = 0.003). No association was found between number of MetSyn components and CES-D score for Buffalo men or women. Similar analyses could not be conducted for Spokane women due to the low number of participants (*n* = 9). Excluding officers with reported CVD or previous treatment for CVD (*n* = 11) did not alter the associations appreciably (data not shown) nor did excluding officers taking antidepressant medications (available for Buffalo police officers only, *n* = 32) (data not shown). Separate analyses excluding Buffalo female officers taking exogenous estrogen (birth control medication: *n* = 20 or hormone replacement medication: *n* = 4) also did not alter the association (data not shown).

### 3.4. Odds Ratios for Individual MetSyn Components by CES-D Score

Odds ratios were calculated for having each individual MetSyn component by CES-D score for Spokane men (Table 4). For each 5-unit increase in CES-D score, the odds of having abdominal obesity increased by 42%. Multivariate adjustment for age, smoking, and marital status slightly attenuated the association (OR = 1.338, 95% CI = 0.932–1.917). For each 5-unit increase in CES-D score, the multivariate adjusted odds increased by 47.6% for having elevated triglycerides (OR = 1.476, 95% CI = 1.025–2.119), by 56.7% for having glucose intolerance (OR = 1.567, 95% CI = 1.056–2.327), and by 51.8% for having hypertension (OR = 1.518, 95% CI = 1.035–2.221).

## 4. Discussion

One surprising finding of this study was the low CES-D scores in all groups (range 5.8–8.7) with between 64.2% and 77.8% of participants scoring in the low category (0–8). This restricted range may have made the finding of a statistical association with MetSyn less likely. However, the prevalence of depression as indicated by a CES-D score ≥16 was comparable with that reported in the Framingham Heart Study (10% in men, 17% in women): 9.1% for Spokane men, 10.2% for Buffalo men, and 16% for Buffalo women, and the prevalence of MetSyn was notably higher for Spokane men and women and Buffalo men (range 31.6%–37.2%) compared to a recent study of US protective service workers (26.1%) [38, 39].

An association between depressive symptoms and number of MetSyn components was observed for male Spokane officers, partially supporting two of the study hypotheses: depression would be associated with a higher number of MetSyn components, and the association would be different between male and female police officers. The association between depressive symptoms and MetSyn found for the Spokane men extends to depressive symptoms in a nonclinical range (i.e., CES-D < 16) [11]. Previous studies have found similar findings in the association between depression and MetSyn among a general population, among young and middle-aged women, among adult male twins, and among those at risk for CVD [11, 13, 22, 26, 28, 40, 41]. One study, noting the peculiarity of their finding, found an association between depressive symptoms and MetSyn in men but not in women [42]. Interestingly, the association found in the current study was significant only among the male officers at one location, Spokane, indicating that there may be potential factors not measured in this study that could have influenced the results, including traditional CVD risk factors such as physical activity and diet, and emerging CVD risk factors like inflammation and work stress [4, 20].

It is noteworthy that the Spokane officers were older and held higher ranks than the Buffalo officers. When the association between depressive symptoms and MetSyn was stratified on these two demographic variables, the association was strongest among the older (>49 years, *n* = 59) versus younger Spokane men (≤49, *n* = 61), and lower-ranking (patrol officer, *n* = 39) versus higher ranking Spokane men (*n* = 81) (data not shown). Yet, due to the small sample sizes for the stratified analyses the findings are reported with caution. Lower ranking police officers may experience high job demand and low job control. Perceived work stress has been associated with depression among older mostly male police officers, and high job strain has been significantly associated with depression among a national sample of working men [2, 43]. Vitaliano et al. suggested that chronic stress leads to depression which increases the risk of MetSyn and subsequently CVD [20].

For Spokane men, the odds of having four of the MetSyn components (abdominal obesity, hypertriglyceridemia, hypertension, glucose intolerance) increased by at least 40% for every 5-unit increase in CES-D score. The association with abdominal obesity was slightly attenuated by adjustment for age, smoking status, and marital status, while HDL-C was not associated with depressive symptoms. This finding is supported by previous studies which have found depression to be associated with abdominal obesity, hypertriglyceridemia, hypertension, and glucose intolerance [11, 19, 29, 40, 44, 45]. The association between depression and MetSyn and its components among these diverse populations suggests that the pathophysiology of both conditions may overlap; yet the biologic mechanisms may vary by MetSyn component. Several potential mechanisms could be considered [46].

Dysfunction of the hypothalamic-pituitary-adrenal (HPA) axis has been the most commonly cited mechanism for the association between depression and MetSyn [29, 46]. Fruehwald-Schultes et al. have shown that hyperinsulinemia activates the HPA axis and increases cortisol secretion [47] and this increase has been associated with depression, the MetSyn, and its individual components [11, 17, 32, 33, 46, 48]. Second, inflammation particularly elevated levels of C-reactive protein (CRP) have been associated with depressive symptoms in younger men, and in elderly men and women [49]. Inflammation has also been acknowledged as a key factor underlying development of MetSyn [22, 46]. Finally, as a means of coping depressed individuals may engage in poor health behaviors, such as a sedentary lifestyle, poor nutrition, cigarette smoking, excessive alcohol intake, or inadequate sleep, and these behaviors may lead to the development of MetSyn [11, 19]. Of these behaviors, only smoking status was available in the current study and it was the Buffalo women who had the highest percent of current and former smokers (57.3%) compared to the other groups (all less than 37%). However, consistent with previous studies, smoking status was included as a potential covariate in multivariate analyses although adjustment attenuated the association minimally across all groups.

Several limitations of the study should be noted. First, the CES-D provides a self-report measure of depressive symptoms as opposed to measuring clinical depression. Underreporting depressive symptoms is of concern with this occupational group as officers may not wish to disclose sensitive information. However, the CES-D has been widely used as a measure for assessing depressive symptoms in epidemiological studies. Second, this study was cross-sectional; therefore, we cannot determine whether depressive symptoms preceded the development of MetSyn and its components or make causal inferences. Others have reported that the MetSyn may lead to depression, specifically that hyperglycemia increases HPA axis activity causing arousal of the central nervous system thereby promoting development of depression [42]. Third, it is possible that participants in these two studies may not be representative of their respective police departments. However, for the Buffalo study comparisons between study participants and the Buffalo, NY Police Department resulted in similar frequency distributions for sex, age, and police rank. Finally, data are from two studies of police officers, thus potentially limiting the generalizability of the study findings to other police departments and other emergency responder populations.

One of the strengths of the current study was the ability to examine the association between depressive symptoms and MetSyn separately for men and women officers. A significant relationship has frequently been found among women with mixed results in men. Female police officers are an understudied occupational group and previous studies of police officers have typically included only male officers. Second, the study population was relatively young (mean age 49 for Spokane, 41 for Buffalo) and we were able to exclude those who have CVD or have received clinical interventions for CVD, enabling us to affirm that the relationship was unlikely attributable to CVD [11]. Third, we were able to exclude officers who were taking antidepressant medications and female officers who reported taking exogenous estrogen, although these exclusions did not alter the study findings. Finally, we used a highly standardized measure as our outcome, MetSyn [37]. In this study MetSyn was defined as the count of the number of components for each individual instead of limiting the assessment to presence/absence of MetSyn for the current study. This approach provides an interpretable measure of association and is more sensitive in the detection of associations [50].

In summary, depressive symptoms were associated with an increasing number of MetSyn components and with higher odds of having the individual MetSyn components among Spokane male police officers. This is the first study that we are aware of which has examined this relationship in police officers. Yet, it is particularly important to understand the association between depression and MetSyn among police officers given the high-stress nature of their job and the higher prevalence of CVD. Future studies should explore potential mediators for this association, including the role of the HPA axis and cortisol, inflammatory processes, and hyperinsulinemia, as these have been cited as potential mechanisms by others. It is also important to explore the gender-specific differences between male and female police officers. Depressive symptomatology and MetSyn are early indicators of future chronic health problems and the results could assist in the development of future gender-specific prevention and intervention efforts among police officers.

## Figures and Tables

**Table 1:** Demographic characteristics by location and gender.

Characteristic	Buffalo New York Police Officers (*N* = 410)				Spokane Heart Study Police Officers (*N* = 130)			
	Men (*N* = 304)		Women (*N* = 106)		Men (*N* = 121)		Women (*N* = 9)	
	*N*	Mean (SD) or %	*N*	Mean (SD) or %	*N*	Mean (SD) or %	*N*	Mean (SD) or %
Age	304	40.8 (7.7)	106	40.8 (5.9)	121	49.1 (6.1)	9	47.3 (2.7)
<40 years	131	43.1	43	40.6	10	8.3	0	0.0
40–49 years	128	42.1	54	50.9	51	42.2	7	77.8
50+ years	45	14.8	9	8.5	60	49.6	2	22.2
Ethnicity								
White	237	79.8	76	71.7	121	100.0	8	88.9
African American	53	17.9	30	28.3	0	0.0	0	0.0
Hispanic	7	2.4	0	0.0	0	0.0	1	11.1
Education								
<12 years, high school/GED	38	12.6	4	3.8	8	6.7	1	11.1
College <4 years	160	53.0	65	61.3	59	49.2	4	44.4
College 4+ years	104	34.4	37	34.9	53	44.2	4	44.4
Marital status								
Single	29	9.6	23	21.7	0	0.0	3	33.3
Married	236	78.2	62	58.5	110	91.7	5	55.6
Divorced	37	12.3	21	19.8	10	8.3	1	11.1
Rank								
Police officer	207	69.5	84	79.3	39	32.2	6	66.7
Sergeant/lieutenant/captain	51	17.1	11	10.4	47	38.8	1	11.1
Detective	34	11.4	9	8.5	28	23.1	2	22.2
Executive	5	1.7	1	0.9	7	5.8	0	0.0
Other	1	0.3	0	0.0	0	0.0	0	0.0
Smoking status								
Current	40	13.2	28	27.2	4	3.3	1	11.1
Former	64	21.2	31	30.1	40	33.1	2	22.2
Never	199	65.7	44	42.7	77	63.6	6	66.7
Body mass index, kg/m^2^	304	30.3 (4.2)	106	26.0 (4.7)	124	29.8 (3.9)	9	28.9 (4.6)
<25	23	7.6	53	50.0	5	4.1	0	0.0
25–30	137	45.1	36	34.0	65	53.7	7	77.8
>30	144	47.4	17	16.0	51	42.2	2	22.2
CES-D score	304	7.5 (6.6)	106	8.7 (8.2)	122	6.6 (5.5)	9	5.8 (4.5)
Low score: 0–8	203	66.8	68	64.2	88	72.7	7	77.8
Middle score: 9–15	70	23.0	21	19.8	22	18.2	2	22.2
High score (depression): 16+	31	10.2	17	16.0	11	9.1	0	0.0

**Table 2:** Mean levels and prevalence of metabolic syndrome by gender and geographic location.

	Component cutpoint (must meet ≥1 of the cutpoints per component)	Prevalence												Mean (SD)			
		Buffalo						Spokane						Buffalo		Spokane	
Metabolic syndrome component		Men		Women		Overall		Men		Women		Overall		Men	Women	Men	Women
		*N*	%*	*N*	%*	*N*	%*	*N*	%*	*N*	%*	*N*	%*	(*N* = 304)	(*N* = 106)	(*N* = 121)	(*N* = 9)
*Abdominal obesity*		**113**	**37.2**	**18**	**17.0**	**131**	**32.0**	**48**	**39.7**	**5**	**55.6**	**53**	**40.8**				
Waist circumference, cm	≥102 Men, ≥88 Women													99.3 (11.4)	79.9 (11.5)	100.3 (10.8)	87.5 (9.9)
*Elevated triglycerides*		**117**	**38.5**	** 9**	** 8.5**	**126**	**30.7**	**52**	**43.0**	**3**	**33.3**	**55**	**42.3**				
Triglycerides, mg/dL	≥150	113	37.2	9	8.5	122	28.6	53	42.7	3	30.0	56	41.8	153.8 (127.4)	88.4 (130.0)	157.6 (102.7)	165.3 (150.6)
Self-reported fibrates or nicotinic acid medication, %	Yes	6	2.0	1	0.9	7	1.7	5	4.1	0	0.0	5	3.8				
*Reduced HDL cholesterol*		**143**	**47.0**	**28**	**26.4**	**171**	**41.7**	**53**	**43.8**	**2**	**22.2**	**55**	**42.3**				
HDL cholesterol, mg/dL	<40 Men, <50 Women	142	46.7	28	24.8	170	40.0	54	43.5	2	20.0	56	41.8	42.2 (12.0)	58.3 (15.7)	42.6 (12.4)	56.0 (16.1)
Self-reported fibrates or nicotinic acid medication, %	Yes	6	2.0	1	0.9	7	1.7	5	4.1	0	0.0	5	3.8				
*Glucose intolerance*		** 83**	**27.3**	**12**	**11.3**	** 95**	**23.2**	**46**	**38.0**	**3**	**33.3**	**49**	**37.7**				
Fasting glucose, mg/dL	≥100	81	26.6	11	10.4	92	21.9	48	38.7	3	30.0	51	38.1	94.7 (14.0)	86.4 (8.4)	98.9 (10.7)	93.4 (10.3)
Self-reported diabetes medication, %	Yes	7	2.3	1	0.9	8	2.0	4	3.3	0	0.0	4	3.1				
*Hypertension*		**129**	**42.4**	**27**	**25.5**	**156**	**38.1**	**55**	**45.5**	**2**	**22.2**	**57**	**43.9**				
Systolic blood pressure, mmHg	≥130	69	22.7	18	17.0	87	20.9	37	29.8	3	30.0	40	29.9	122.2 (11.3)	116.7 (13.3)	125.0 (10.3)	121.8 (10.1)
Diastolic blood pressure, mmHg	≥85	79	26.0	17	16.0	96	23.3	35	28.2	0	0.0	35	26.1	78.7 (10.2)	74.6 (9.6)	81.1 (8.5)	78.9 (4.7)
Self-reported hypertension with antihypertensive medication, %	Yes	45	14.8	7	6.6	52	12.7	17	14.0	0	0.0	17	13.0				
*Number of components*														**1.9 (1.4)**	**0.9 (1.1)**	**2.1 (1.4)**	**1.7 (1.9)**
	0	54	17.8	53	50.0	107	26.1	14	11.6	4	44.4	18	13.9				
	1	75	24.7	25	23.6	100	24.4	32	26.5	1	11.1	33	25.4				
	2	79	26.0	19	17.9	98	23.9	30	24.8	1	11.1	31	23.9				
	3	48	15.8	6	5.7	54	13.2	22	18.2	1	11.1	23	17.7				
	4	32	10.5	2	1.9	34	8.3	19	15.7	1	11.1	20	15.4				
	5	16	5.3	1	0.9	17	4.2	4	3.3	1	11.1	5	3.9				
	**Prevalence (≥3)**	** 96**	**31.6**	** 9**	** 8.5**	**105**	**25.6**	**45**	**37.2**	**3**	**33.3**	**48**	**36.9**				

**Table 3:** Unadjusted and adjusted number of metabolic syndrome components by categories of CES-D score by location and gender.

Categories of CES-D	Number of metabolic syndrome components								
	Unadjusted			Age-adjusted			Multivariate adjusted*		
	*N*	Mean (SD)	*P* value**	*N*	Mean (SD)	*P* value**	*N*	Mean (SD)	*P* value**
*Buffalo men*									
0–8	203	1.8 (1.4)		203	1.8 (0.1)		202	1.9 (0.1)	
9–15	70	2.2 (1.3)	0.431	70	2.2 (0.2)	0.379	69	2.3 (0.2)	0.390
≥16	31	1.9 (1.5)		31	2.0 (0.2)		31	2.0 (0.3)	
*Buffalo women*									
0–8	68	0.8 (1.1)		68	0.8 (0.1)		65	0.8 (0.1)	
9–15	21	1.1 (1.4)	0.376	21	1.1 (0.2)	0.377	21	1.2 (0.3)	0.610
≥16	17	0.9 (0.9)		17	0.9 (0.3)		17	0.7 (0.3)	
*Spokane men*									
0–8	88	1.9 (1.3)		88	1.9 (0.1)		88	1.8 (0.3)	
9–15	22	2.6 (1.3)	0.003	22	2.6 (0.3)	0.004	21	2.7 (0.4)	0.003
≥16	11	3.1 (1.6)		11	3.0 (0.4)		11	3.0 (0.5)	

*Adjusted for age, smoking status, and marital status.

**P values are based on the continuous CES-D score.

**Table 4:** Unadjusted and adjusted odds ratios* for MetSyn components by CES-D score for Spokane men.

MetSyn component	Unadjusted (*N* = 121)		Age-adjusted (*N* = 121)		Multivariate adjusted** (*N* = 120)	
	Odds ratio	95% CI	Odds ratio	95% CI	Odds ratio	95% CI
Abdominal Obesity	1.422	1.010–2.003	1.396	0.985–1.977	1.338	0.932–1.917
Elevated Triglycerides	1.364	0.970–1.917	1.449	1.020–2.064	1.476	1.025–2.119
Reduced HDL-C	0.975	0.699–1.357	1.010	0.718–1.416	1.030	0.730–1.456
Glucose Intolerance	1.553	1.088–2.202	1.456	1.005–2.119	1.567	1.056–2.327
Hypertension	1.396	0.985–1.968	1.370	0.965–1.942	1.518	1.035–2.221

*Odds ratios for having each metabolic syndrome component are based on a 5-unit change in CES-D score.

**Adjusted for age, smoking status, and marital status.
